# Individualized scan protocols for CT angiography: an animal study for contrast media or radiation dose optimization

**DOI:** 10.1186/s41747-023-00332-1

**Published:** 2023-04-26

**Authors:** Johannes Haubold, Sebastian Zensen, René Hosch, Benedikt Michael Schaarschmidt, Denise Bos, Bernhardt Schmidt, Thomas Flohr, Yan Li, Michael Forsting, Hubertus Pietsch, Felix Nensa, Gregor Jost

**Affiliations:** 1 Department of Diagnostic and Interventional Radiology and Neuroradiology, University Hospital Essen, 45147 Essen, Germany; 2 Institute of Artificial Intelligence in Medicine, University Hospital Essen, Essen, Germany; 3 MR and CT Contrast Media Research, Bayer AG, Berlin, Germany; 4 Siemens Healthcare GmbH, Forchheim, Germany

**Keywords:** Animals, Contrast media, Radiation, Tomography (x-ray computed)

## Abstract

**Background:**

We investigated about optimization of contrast media (CM) dose or radiation dose in thoracoabdominal computed tomography angiography (CTA) by automated tube voltage selection (ATVS) system configuration and CM protocol adaption.

**Methods:**

In six minipigs, CTA-optimized protocols were evaluated regarding objective (contrast-to-noise ratio, CNR) and subjective (6 criteria assessed by Likert scale) image quality. Scan parameters were automatically adapted by the ATVS system operating at 90-kV semi-mode and configured for standard, CM saving, or radiation dose saving (image task, quality settings). Injection protocols (dose, flow rate) were adapted manually. This approach was tested for normal and simulated obese conditions.

**Results:**

Radiation exposure (volume-weighted CT dose index) for normal (obese) conditions was 2.4 ± 0.7 (5.0 ± 0.7) mGy (standard), 4.3 ± 1.1 (9.0 ± 1.3) mGy (CM reduced), and 1.7 ± 0.5 (3.5 ± 0.5) mGy (radiation reduced). The respective CM doses for normal (obese) settings were 210 (240) mgI/kg, 155 (177) mgI/kg, and 252 (288) mgI/kg. No significant differences in CNR (normal; obese) were observed between standard (17.8 ± 3.0; 19.2 ± 4.0), CM-reduced (18.2 ± 3.3; 20.5 ± 4.9), and radiation-saving CTAs (16.0 ± 3.4; 18.4 ± 4.1). Subjective analysis showed similar values for optimized and standard CTAs. Only the parameter diagnostic acceptability was significantly lower for radiation-saving CTA compared to the standard CTA.

**Conclusions:**

The CM dose (-26%) or radiation dose (-30%) for thoracoabdominal CTA can be reduced while maintaining objective and subjective image quality, demonstrating the feasibility of the personalization of CTA scan protocols.

**Key points:**

• Computed tomography angiography protocols could be adapted to individual patient requirements using an automated tube voltage selection system combined with adjusted contrast media injection.

• Using an adapted automated tube voltage selection system, a contrast media dose reduction (-26%) or radiation dose reduction (-30%) could be possible

## Background

The continuously increasing number of computed tomography (CT) scans worldwide and the accompanying exposure to radiation and iodinated contrast media (CM) [1] are not equally relevant for all patients. In younger patients or in patients with an adequate renal function that require repeated/regular follow-up examinations, the CM dose appears to be less concerning than the radiation exposure to reduce the increase in lifetime attributable cancer risk due to the radiation exposure. In the elderly and especially in patients with chronic kidney disease, however, the possible risks of contrast administration may outweigh those associated with radiation, although the impact of intravenous CM administration in patients with chronic kidney disease is discussed controversially [2].

Several approaches for reducing CM dose have been established in recent years [3–6]. For CT angiography (CTA), the contrast-to-noise ratio (CNR) between vasculature and surrounding tissue is the most important image quality parameter. Both the contrast media and radiation dose have a direct impact on the CNR. In theory, an increase of contrast media dose or contrast media attenuation allows for higher image noise levels while maintaining vascular CNR. In turn, image noise is inversely correlated with the square root of the radiation dose [7]. Taking this together and assuming an unchanged vascular CNR, an increasing contrast media dose or attenuation allows for a reduction of radiation dose and vice versa. This general concept also applies to low-kV CTA, where the attenuation of iodinated CM can be increased up to a factor of 2 if the tube voltage is lowered compared to the standard setting of 120 kV [8]. With this approach, CM dose can be reduced while maintaining image quality and radiation dose [9]. However, in the vast majority of low-kV CTA applications, both parameters, CM dose and radiation dose, have been reduced [10–12]. This requires careful parameter optimization to maintain image quality, in particular regarding radiation and nonlinear correlations to CNR. Low-kV CT may also lead to higher image noise due to lower tissue penetration, especially in obese patients [13]. This can be compensated to some extent by using high-power x-ray tubes, but the iodine attenuation also decreases with larger patient diameters [14, 15].

Automated tube voltage selection (ATVS) systems can optimize tube voltage and mAs output to minimize radiation dose while maintaining image quality in terms of CNR in a personalized way by considering the attenuation profile of the patient [16]. In the case of CTA, the ATVS optimization is based solely on the higher iodine CNR at lower tube voltages and relies on the assumption that the CM injection protocol remains unchanged. Two parameters of the ATVS system have to be configured: first, adjustment of the image quality level by specifying an image quality reference (tube voltage/mAs output) and, second, setting of a specific imaging task.

Besides its intended use, this ATVS system configuration also has the potential to optimize CTA scans for a minimal CM dose or minimal radiation exposure based on the general concept introduced above. For this approach, the image task and reference setting have to be combined with an adjusted CM injection protocol. A reduction of the ATVS slider setting (to the parenchymal or unenhanced imaging task) results in a reduction of image noise by increasing the radiation dose, which in turn allows a reduction of the CM dose at constant CNR. On the other hand, the ATVS reference settings can be optimized for image quality to reduce the radiation dose. The resulting higher image noise can be compensated by increasing the CM dose.

The primary aim of this experimental CTA study was to investigate the feasibility of a combined ATVS system and injection protocol approach for either minimal radiation dose or minimal contrast dose. The secondary aim was to evaluate the feasibility of this optimization approach under obesity conditions. In all studies, objective and subjective image quality ratings were used as evaluation metrics.

## Methods

### Study design

**Fig. 1:**
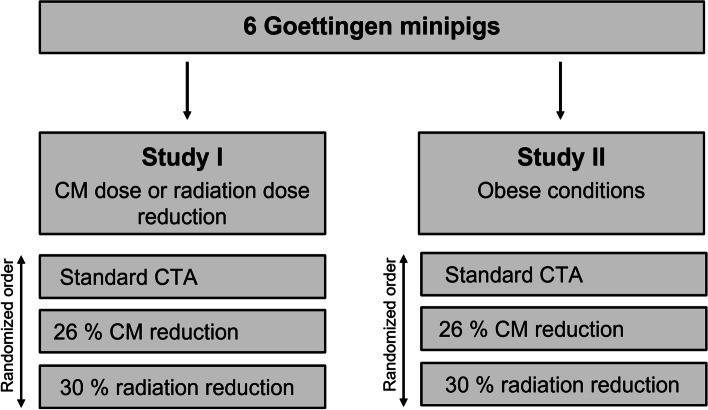
Schematic illustration of the workflow of studies 1 and 2

This study on six healthy minipigs was divided into two sub-studies (Fig. 1). Study 1 aimed to optimize a standard CTA for either a minimum radiation exposure or a minimum contrast dose (primary aim). Study 2 evaluated the optimization of radiation exposure or contrast dose under obesity conditions (secondary aim). The three CTA protocols of each sub-study were examined in one scanning session in randomized order with a 45-min CM washout period between examinations. The topogram was not repeated between the examinations to avoid any effect on dose modulation. The bladder was not in the scan area to avoid artifacts due to the high density of excreted CM.

### Animals

The study was performed on six Goettingen minipigs (Ellegaard, Dalmose, Denmark) weighting 38.7 ± 4.9 kg (mean ± standard deviation [SD]) with an average abdominal cross-section of 27.0 ± 2.6 cm at liver level. The animals were handled in compliance with the German Animal Welfare Legislation and with the approval of the State Animal Welfare Committee. CT was performed under general anesthetic induced with intramuscular injection of 15 mg/kg ketamine (Pharmacia, Karlsruhe, Germany), 2 mg/kg azaperone (Stresnil, Elanco GmbH, Bad Homburg, Germany), and 0.02 mg/kg atropine (Eifelfango Chem.-Pharm. Werke, Bad Neuenahr-Ahrweiler, Germany). After intravenous application of 7 mg/kg propofol (Propofol-Lipuro, Braun, Melsungen, Germany), they were orally intubated and ventilated with an air/oxygen mixture. Anesthesia was maintained with 12 mg/kg/h propofol. CTA was performed in a prone position during end-expiratory ventilation stop. Heart rate and oxygen saturation were monitored. For study 2, obesity was simulated by placing 12 saline bags (1000 mL Ecoflag, B. Braun Melsungen AG, Melsungen, Germany) around the animal, increasing the effective cross section by about 30%.

### CT device, technical settings, and CM

All CTA scans were performed on a 192-slice dual-source CT (Somatom Force, Siemens Healthineers, Erlangen, Germany) with 192 × 0.6-mm collimation, 500-mm scan length, 0.5-s rotation time, and 1.2 pitch, resulting in a scan time of 4.3 s. For automated exposure control, automated tube current modulation was applied in combination with an ATVS system (CAREkV, Siemens Healthineers, Erlangen, Germany) [16] operated in 90-kV semi-mode. Image reconstruction was performed using a Bv36 kernel and SAFIRE level 3 using a field of view of 350 × 350 mm^2^ and a slice thickness of 0.75 mm with 0.5-mm increment between slices. As CM, Iopromide 300 (Ultravist 300, Bayer Vital GmbH, Leverkusen, Germany) was used. All contrast injections were performed with an injection system (Medrad Centargo, Bayer AG, Berlin, Germany) and were followed by a 20-ml saline flush. Bolus tracking (descending aorta, trigger level = 100 HU, trigger delay = 3 s) was used for correct scan timing. Radiation exposure for each scan was assessed as volume-weighted CT dose index (CTDI_vol_). The detailed parameters of the ATVS setting and the respective injection protocols for each study are listed in Table 1.

**Table 1:** ATVS parameters, radiation dose, and contrast media protocols

Study 1	Standard CTA	CM reduction	Radiation reduction
ATVS and radiation dose	CAREkV 90-kV semi-mode		
Reference (kV/mAs)	120/120	120/120	120/84
Slider position	11	3	11
CTDI_vol_ (mGy)	2.4 ± 0.7	4.3 ± 1.1	1.7 ± 0.5
CM protocol			
Concentration (mgI/mL)	Iopromide 300	Iopromide 300	Iopromide 300
CM dose (mgI/kg)	210	155	252
CM volume (mL)	27.2 ± 3.5	20.2 ± 2.8	32.2 ± 4.2
Flow rate (mL/s)	3.5	2.6	4.2
IDR (g I/s)	1.1	0.8	1.3
Study 2	Standard CTA	CM reduction	Radiation reduction
ATVS and radiation dose	CAREkV 90-kV semi-mode		
Reference (kV/mAs)	120/120	120/120	120/84
Slider position	11	3	11
CTDI_vol_ (mGy)	5.0 ± 0.7	9.0 ± 1.3	3.5 ± 0.5
CM protocol			
Concentration (mgI/mL)	Iopromide 300	Iopromide 300	Iopromide 300
CM dose (mgI/kg)	240	177	288
CM volume (mL)	31.1 ± 4.0	23.0 ± 3.2	36.8 ± 4.9
Flow rate (mL/s)	4.0	3.0	4.8
IDR (g I/s)	1.2	0.9	1.4

*ATVS* Automated tube voltage selection, *CM* Contrast media, *CTA* Computed tomography angiography, *IDR* Iodine delivery rate

### Objective measurement of image quality

**Fig. 2:**
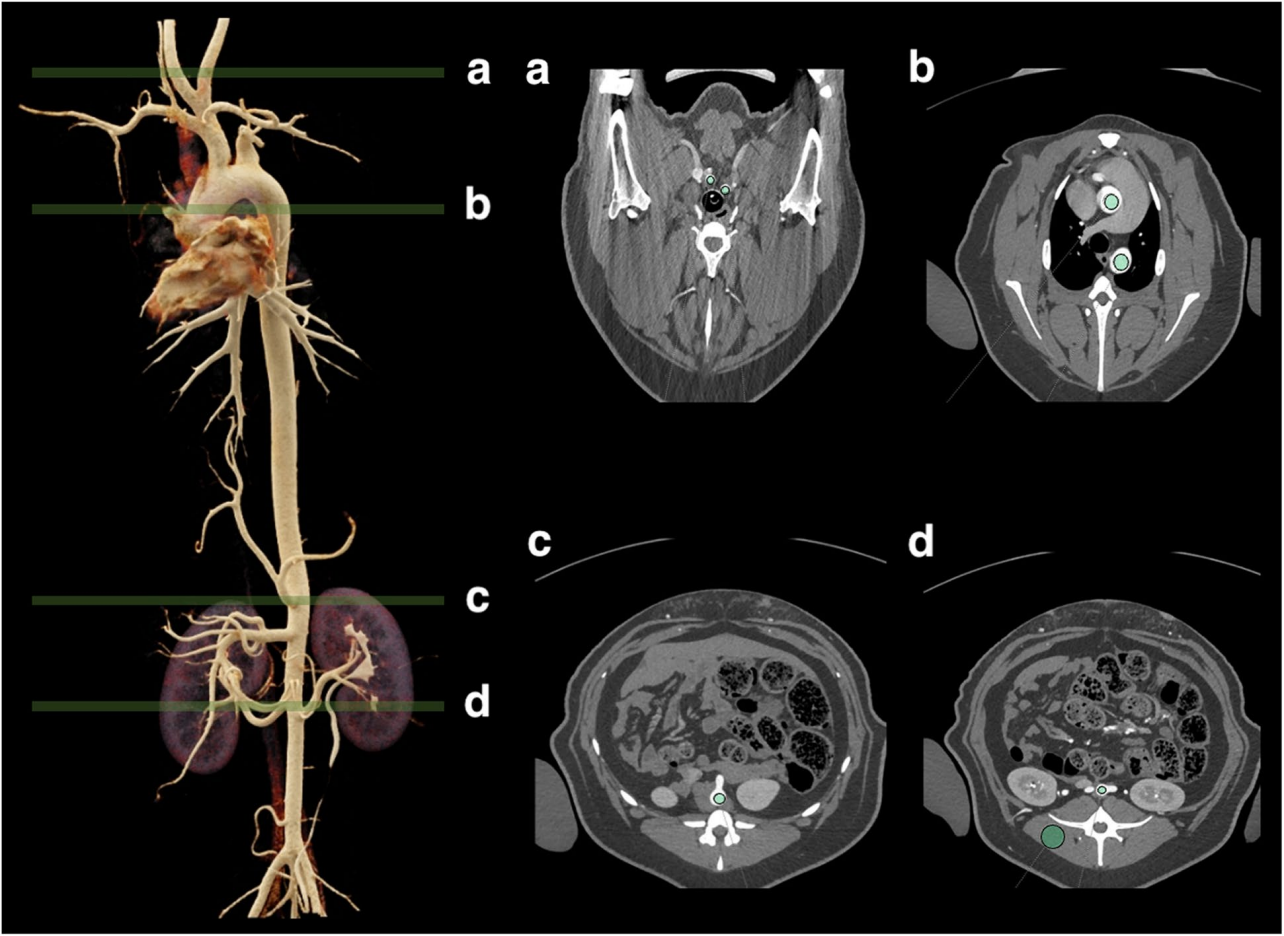
Left: three-dimensional visualization by cinematic rendering of the vascular anatomy with axial planes for ROI measurements. Right: exemplary region-of-interest-based measurements in the common carotid artery on both sides (**a**), in the ascending and descending aorta (**b**), in the abdominal aorta at the level of the celiac trunk, and in the abdominal aorta and autochthonous back muscles at the level of the renal arteries

To analyze quantitative image quality, signal attenuation was measured standardized at multiple regions of interest (ROIs): left and right common carotid artery, ascending and descending thoracic aorta, and abdominal aorta at the level of the celiac trunk and at the level of the renal arteries (Fig. 2). Additionally, the signal attenuation and standard deviation of the autochthonous muscle (level of the renal arteries) were determined. Subsequently, the CNR was calculated as follows:

$$\mathrm{CNR}=\left(\mathrm{attenuation vessel}-\mathrm{attenuation muscle}\right)/\mathrm{SD muscle}$$

In addition, for quantitative image similarity analysis between standard and CM or radiation-optimized protocols, the Fréchet inception distance (FID) [17], L1 error [18], and peak signal-to-noise ratio (PSNR) [19] were calculated. The FID was proposed by Heusel et al. [17] and is using the Inception V3 network to compare the distribution of activations of two image groups (real and generated) using the Wasserstein distance. A low FID indicates a similar distribution of the two image groups [17]. The L1 error, on the other hand, denotes the absolute deviation between the real and generated image. Therefore, a low L1 indicates a lower difference between the real and generated image [18]. In addition, another common metric is the PSNR which can be understood as a pixel-based mean-squared error between two images (real and generated) [19]. In opposite to the FID, a high PSNR value corresponds with higher image quality.

### Subjective measurement of image quality

The blinded CT examinations were independently assessed by two board-certified radiologists with 10 and 11 years of experience in CT imaging. Six different aspects were evaluated with a 5-point Likert scale: image quality/diagnostic acceptability, sharpness, image contrast, image noise, artifacts, and visibility of the branches of the renal artery (Table 2).

**Table 2:** Qualitative image quality assessment criteria

Score	Image quality — diagnostic acceptability	Sharpness	Image contrast	Image noise	Artefacts	Visible branches of the renal artery
5	5 = excellent	5 = sharpest	5 = excellent image contrast	5 = minimal image noise	5 = no artifacts	5 = sharpest
4	4 = completely acceptable	4 = better than acceptable	4 = above average contrast	4 = less than average noise	4 = minor artifacts not interfering with diagnostic decision-making	4 = better than average
3	3 = mostly acceptable	3 = acceptable	3 = acceptable image contrast	3 = average image noise	3 = acceptable artifacts	3 = average
2	2 = only acceptable under limited conditions	2 = suboptimal	2 = suboptimal image contrast	2 = above average noise	2 = major artifacts affecting visualization of major structures but diagnosis still possible	2 = poorer than average
1	1 = unacceptable	1 = blurry	1 = very poor contrast	1 = unacceptable image noise	1 = artifacts affecting diagnostic information	1 = blurry and unacceptable

### Statistical analysis

All results are presented as mean ± SD or median with interquartile range for the subjective image quality rating. Cohorts were tested for normality using the Shapiro–Wilk test. Two-way ANOVA with ROI and group variables with paired data was applied to compare CNR measurements of CM, and radiation reduced images with standard CTA images. Friedman test followed by Dunn multiple comparison was applied to evaluate the subjective image quality analysis. A *p*-value of less than 0.05 has been considered statistically significant. Statistical analyses were performed with Prism 7 (GraphPad Software, San Diego, USA).

## Results

### Study 1: optimization of radiation dose or contrast dose

#### Radiation dose, noise, and attenuation

**Fig. 3:**
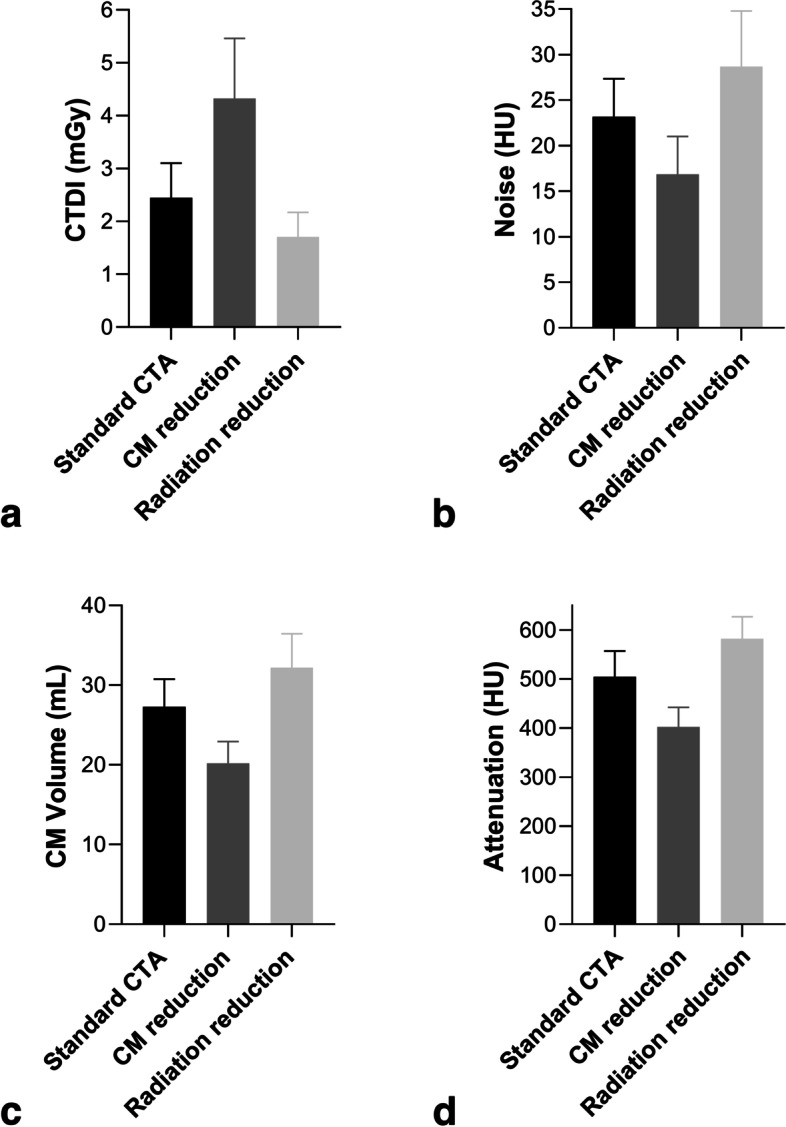
Volume-weighted computed tomography dose index (**a**), image noise (**b**), contrast media volume (**c**), and vascular attenuation (**d**) of study 1, shown as mean ± standard deviation

The ATVS settings for the standard CTA resulted in a CTDI_vol_ of 2.4 ± 0.7 mGy. For the CTAs with reduced CM dose, CTDI_vol_ was on average 77% higher, and optimization of ATVS settings for low radiation dose resulted in a 30% CTDI_vol_ reduction (Fig. 3a). These differences in radiation dose resulted in decreased noise levels (-23%) for CM-reduced CTAs and increased image noise (+ 29%) for the radiation-reduced scans (Fig. 3b). The reference CM dose of 210 mgI/kg body weight resulted in a CM volume of 27.2 ± 3.5 mL. The adapted CM protocols had a 26% lower volume for CM-optimized CTAs and a 30% higher volume for low radiation exams (Fig. 3c). The resulting vascular attenuation — averaged across all ROIs — was 504.4 ± 52.8 HU for the standard CTA, 402.1 ± 52.8 HU (-20%) for the CM-reduced CTAs, and 582.3 ± 44.7 HU (+ 15%) for the CTAs with a reduced radiation dose (Fig. 3d).

### Objective image quality

**Fig. 4:**
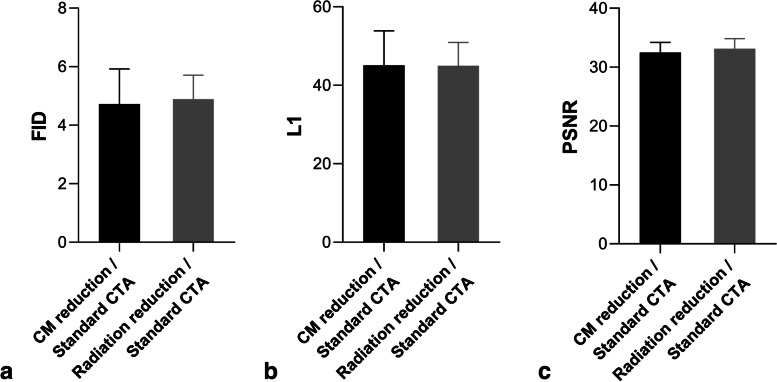
Image similarity parameter Fréchet inception distance (**a**), L1 (**b**), and peak signal-to-noise ratio (**c**) shown as mean ± standard deviation

**Fig. 5:**
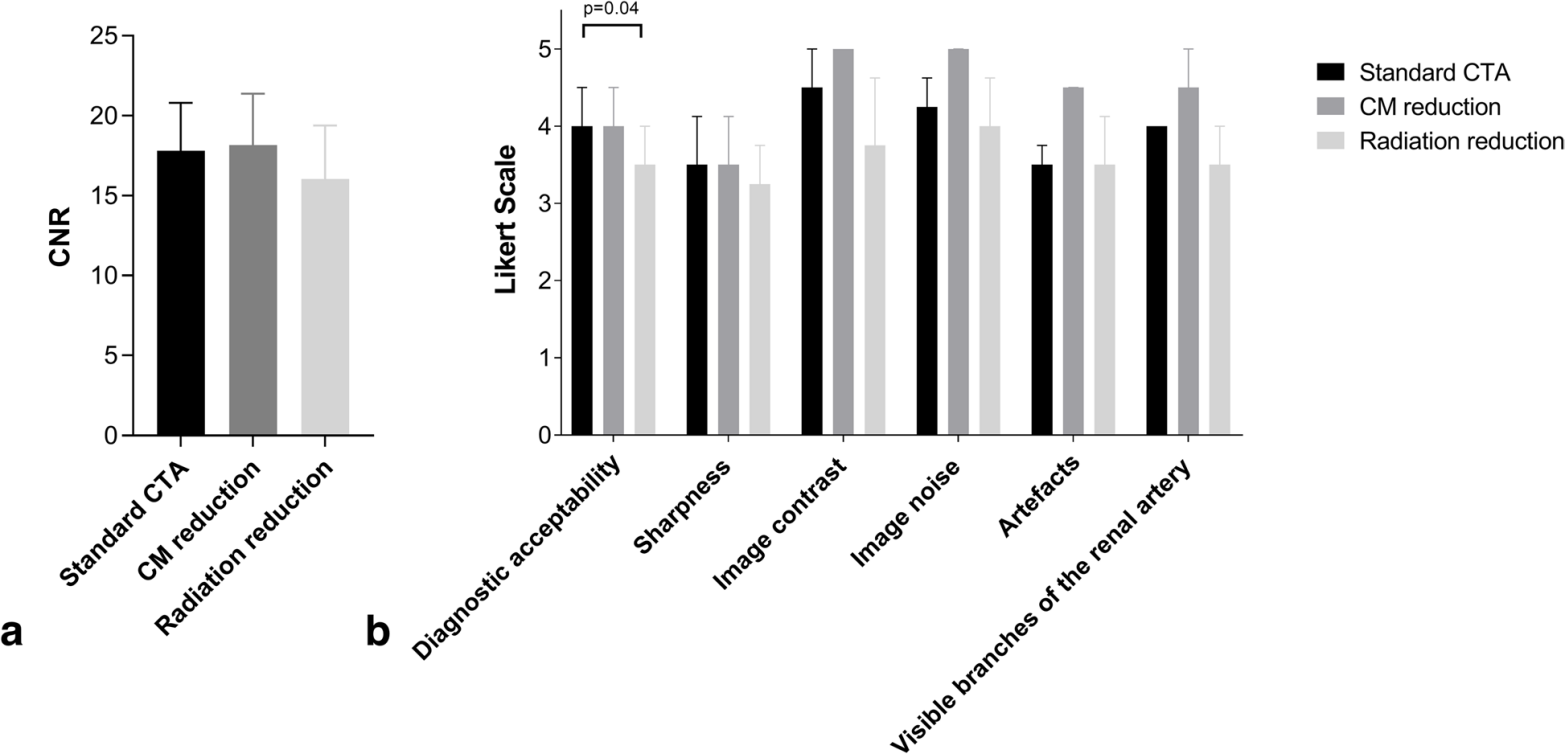
Contrast-to-noise ratio values of the three groups, shown as mean ± standard deviation (**a**). Subjective image quality characteristics of the three groups, shown as median and interquartile range (**b**)

The image similarity parameters (FID, L1, PSNR) revealed high similarity between the CM dose- and radiation dose-optimized CTA images compared to the standard CTA (Fig. 4). The CNR was comparable between all groups, although the CTAs optimized for radiation reduction showed a small but nonsignificant reduction (16.0 ± 3.4) compared to the almost identical CNR in the standard CTA (17.8 ± 3.0) and CM-reduced CTA (18.2 ± 3.3) (Fig. 5a).

### Subjective image quality

Subjective image quality analysis revealed consistently good to excellent Likert scale ratings for all groups. The only significant difference was a reduced rating in diagnostic acceptability for the radiation-optimized CTA images compared to the standard CTA (*p* = 0.042) (Fig. 5b).

**Fig. 6:**
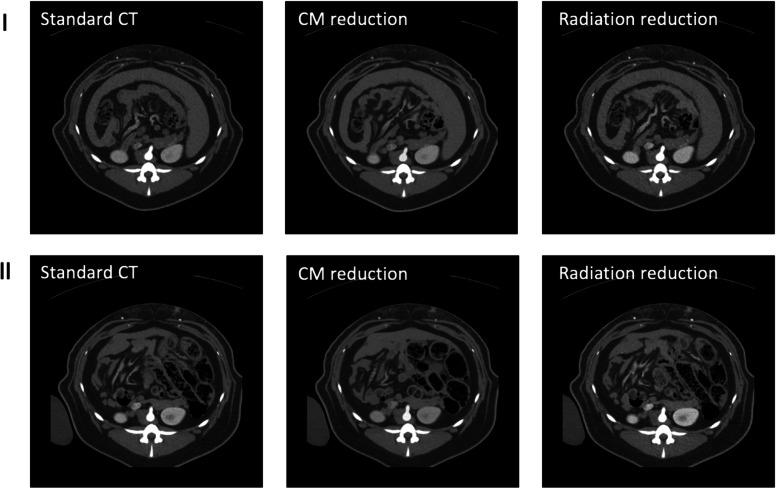
Exemplary images of a slice at the level of the celiac trunk of animal 1 of the three groups (standard computed tomography, contrast medium reduction, and radiation reduction) in studies 1 and 2

Representative images from animal 1 at the level of the celiac trunk are shown in Fig. 6. The results of CNR measurements and similarity analyses of both studies are shown in Table 3.

**Table 3:** Quantitative and qualitative image quality parameters

Study 1	Standard CTA	CM reduction		Radiation reduction	
Quantitative image quality (mean ± SD)					
CNR	17.8 ± 3.0	18.2 ± 3.3	*p* = 0.889^1^	16.0 ± 3.4	*p* = 0.070^2^
Similarity *versus* standard CTA (mean ± SD)					
PSNR	−	32.5 ± 1.7		33.2 ± 1.7	
FID	−	4.7 ± 1.2		4.9 ± 0.8	
L1	−	45.1 ± 8.8		45.0 ± 6.0	
Subjective image quality (median (IQR))					
Diagnostic acceptability	4 (4–4)	4.5 (4–5)	*p* > 0.999^1^	3 (2.3–4)	*p* = 0.042^2^
Sharpness	4 (4–5)	5 (4–5)	*p* > 0.999^1^	4 (3.3–4)	*p* = 0.560^2^
Image contrast	3.5 (3–4.8)	4.5 (4–5)	*p* = 0.582^1^	4 (3–4.8)	*p* = 0.582^2^
Image noise	4 (3.3–4)	5 (4–5)	*p* = 0.582^1^	3 (3–4)	*p* > 0.999^2^
Artifacts	4 (4–4)	4 (4–5)	*p* = 0.091^1^	3.5 (3–4)	*p* > 0.999^2^
Visible branches of renal artery	5 (4–5)	5 (4–5)	*p* = 0.130^1^	5 (4–5)	*p* = 0.745^2^
Study 2	Standard CTA	CM reduction		Radiation reduction	
Quantitative image quality (mean ± SD)					
CNR	19.2 ± 4.0	20.5 ± 4.9	*p* = 0.325^1^	18.4 ± 4.1	*p* = 0.600^2^
Similarity *versus* standard CTA (mean ± SD)					
PSNR	-	31.6 ± 2.6		30.7 ± 2.3	
FID	-	6.3 ± 0.9		4.9 ± 0.8	
L1	-	49.6 ± 10.7		56.0 ± 10.8	
Subjective image quality (median (IQR))					
Diagnostic acceptability	4 (4–4.8)	4.5 (4–5)	*p* = 0.100^1^	4 (3–4)	*p* = 0.978^2^
Sharpness	5 (4–5)	5 (4–5)	*p* = 0.305^1^	5 (4.3–5)	*p* > 0.999^2^
Image contrast	5 (4.3–5)	5 (4.3–5)	*p* = 0.379^1^	5 (4.3–5)	*p* > 0.999^2^
Image noise	4 (3–4)	4 (3–4.8)	*p* = 0.131^1^	3.5 (3–4)	*p* = 0.899^2^
Artifacts	3.5 (3–4)	4 (4–5)	*p* = 0.027^1^	4 (3–4)	*p* > 0.999^2^
Visible branches of renal artery	5 (4.3–5)	5 (4.3–5)	*p* = 0.341^1^	5 (4.3–5)	*p* > 0.999^2^

^1^CM reduction *versus* standard CTA

^2^Radiation reduction *versus* standard CTA

*ATVS* Automated tube voltage selection, *CM* Contrast media, *CNR* Contrast-to-noise ratio, *CTA* Computed tomography angiography, *FID* Fréchet inception distance, *PSNR* Peak signal-to-noise ratio, *SD* Standard deviation, *IQR* Interquartile range

#### Study 2: protocol optimization under obesity conditions

To investigate the influence of obesity on the presented approach of radiation exposure or contrast dose optimization, obese conditions were simulated by increasing the cross section of the animals by adding saline bags. The respective *CTDI*_vol_ was 5.0 ± 0.7 mGy (standard CTA), 9.0 ± 0.7 mGy (CM reduction), and 3.5 ± 0.5 mGy (radiation reduction).

### Objective image quality

The image similarity parameters L1 and PSNR showed high similarity of the CTA procedures optimized for CM or radiation dose with the standard CTA (Table 3). The mean CNR and its SD were very similar of the standard CTA in comparison with the two CTA optimization approaches with no significant differences (standard CTA, 19.2 ± 4.0; CM reduction, 20.5 ± 4.9; radiation reduction, 18.4 ± 4.1).

### Subjective image quality

The subjective image quality analysis showed good to excellent results for all groups. Small but statistically significant differences were detected for the presence of artifacts, whereby a higher Likert score was found for the CM dose reduction group compared to the standard CTA (*p* = 0.027) (Table 3).

## Discussion

The aim of the study was to optimize scanning and injection protocols in thoracoabdominal CTA without affecting image quality, using a combination of ATVS settings and injection protocols. For this purpose, two sub-studies were performed: study 1: optimization for minimal radiation or CM dose and study 2: protocol optimization under obesity conditions. A 90-kV scan protocol with kV-adapted CM dose served as the reference standard. The protocols were optimized for one factor at a time, e.g., the lowest possible contrast dose. To achieve this, the radiation dose determined by the ATVS settings was adjusted to theoretically achieve the same vascular CNR.

Our experimental study demonstrated that a combination of ATVS settings and adapted CM injection protocols can be used to significantly reduce either the CM dose or the radiation dose in thoracoabdominal CTA without affecting image quality.

The quantitative results confirmed our optimization approach. Very similar vascular CNR values were achieved for the three CTA protocols in study 1, with either a 26% CM reduction or a 30% radiation dose reduction with the same image quality regarding CNR. Consistent with the aforementioned results, the evaluation of the similarity analysis (FID, L1, and PSNR) demonstrated a high level of similarity to the reference protocols.

Additionally, five of six evaluated subjective image quality parameters revealed similar ratings for the standard CTA, the exam optimized for CM dose reduction, and the radiation-reduced scans. Only the parameter diagnostic acceptability was rated significantly lower for the radiation dose-optimized images. Nevertheless, this still averaged a score above 3, which is considered diagnostically acceptable. A possible reason for the difference between the CNR evaluation and the diagnostic acceptability ratings could be the different weighting of image noise between objective and subjective image quality. Changing the ATVS slider setting changes the radiation dose; accordingly, a low slider setting reduces the image noise by increasing the dose. While the mean CNR is very similar between the groups, the higher noise level (higher ATVS setting and lower reference mAs) in the images with reduced radiation dose could be responsible for a lower subjective evaluation of diagnostic acceptability.

Our results confirm and extend a recent CTA study demonstrating that radiation or CM dose optimization — by combining scan and contrast protocols — can result in a comparable image quality to a 120-kV reference exam [20]. In the study, a reduction in the tube voltage from 120 to 90 kV without adjusting the CM dose led to a reduction in the radiation dose of about 34%. In a second 90-kV exam, the CM dose was reduced by about 20%, and the ATVS imaging task slider was set from level 11 to level 3. This leads to an optimization of image noise by increasing the radiation dose. Overall, this change in the ATVS slider setting keeps the CNR constant while reducing the contrast media dose. It results in a similar quantitative and qualitative image quality as the 120-kV exam. Our study goes a step further and demonstrates that the CTA optimization approach is independent of the kV setting for both CM and radiation dose optimization. It thus complements the results of Martens et al. [21], showing that the combination of ATVS settings and CM injection parameters can be used to optimize the radiation dose or amount of CM for parenchymal liver CT at identical kV. In comparison, greater savings were obtained in CM (-26% *versus* -16%) or radiation dose (-30% *versus* -26%) for the vascular contrast in our study.

In doing so, it complements the results of the volcanic study, which recently demonstrated for CT coronary angiography the CM savings that can be achieved by kV adjustments in 10-kV steps at constant CNR [12]. Nevertheless, it also highlights the nonlinear correlation between noise and radiation dose [22]. With a 26% reduction in the amount of CM, the radiation dose had to be increased by 77% to keep the CNR constant. This, of course, rather limits the field of application to elderly renal insufficient patients who would be less affected by possible long-term consequences of radiation exposure. Overall, however, elderly also belong to the group of patients most frequently affected by chronic kidney damage [23].

The efficacy of our protocol optimization approach was also tested under simulated obesity conditions, which frequently occur in the thoracoabdominal scan region. Similar results in CNR evaluation validated the quantitative comparability of the optimized protocols in relation to the standard CTA. Subjective image analysis also showed good to very good results on the Likert scale for all evaluation criteria and groups. Compared to the standard CTA, no significantly lower ratings were found neither for the CM nor for the radiation-reduced images. The results are in line with current data on CT coronary angiography in obese patients [24, 25]. To reduce noise levels [26] in patients with a high body mass index, it is often recommended to increase the tube voltage. However, our experimental data suggest that a reduction in radiation dose or contrast dose using ATVS may also be feasible under obesity conditions at low tube voltages. The experimental obesity conditions result in a doubling of the absolute radiation dose; however, a radiation reduction of 30% was still achievable compared to the standard protocol — the same level of reduction was demonstrated for normal body size conditions. In our study, obesity was simulated by adding saline bags to the radiation field, leading to an object cross section of about 30 by 40 cm. This does not represent the full range of obesity levels observed in patients, as the animals more closely resemble children rather than adults in terms of body weight (averaging just under 40 kg), and the cross section in the thoracoabdominal scan region (average 27 cm) also more closely resembles a slim adult. Although pigs with saline bags do not equate to humans, multiple studies have shown that increased diameter of water phantoms can produce similar attenuation to obese humans [27]. Thus, the experimental setup in this study demonstrates that the basic principle also works for larger diameters as in overweight adults.

The proposed approach to optimize CTA examinations is based on the combination of radiation exposure and CM dose to keep the vascular CNR constant. Both parameters can be calculated for each individual patient, and the settings for the CTA scan and injector protocol can be adapted manually. In our study, the ATVS system was used beyond the vendor-recommended application to automatically individualize the radiation dose for the patient and the specific optimization task. A further extension of the ATVS system holds the potential for user-friendly personalization of CTA exams by considering patient-specific factors such as age or renal status and CM protocol settings as iodine dose and flow rate.

Our study demonstrates the feasibility of scan protocol personalization in an animal model. Although minipigs are an established model that resembles the human anatomy in many aspects, they differ from human patients, particularly in anatomy and size. For instance, the abdominal cross section of the animals (27 cm) is at the lower end of the abdominal diameters reported in human clinical imaging studies (25–42 cm [28] or 28 cm [29]). For study 2, we significantly increased the effective diameter of the animals by placing saline bags on them and were able to show that the concept works just as well under these conditions.

The study was conducted with only a small group of six animals. However, intraindividual comparison under highly standardized conditions is a powerful method to obtain reliable results with a small number of subjects. This is especially true for the quantitative evaluation. Subjective qualitative evaluation methods, however, have only a low significance with this small sample number, so that the effects on subjective image quality cannot be conclusively clarified. Nevertheless, even though the three protocols correspond to nearly identical CNR, they differ in terms of vascular attenuation and image noise. Therefore, qualitative evaluation is important to estimate subjective image quality and diagnostic acceptability.

Another limitation is the restriction of the ATVS system to 90 kV. In our study, the x-ray tube power was sufficient to adapt to meet the higher demands on obese patients, but this may not be the case in all clinical situations or with other CT systems. Other tube voltages might be considered for other patient groups or indications. This in turn would also require an adaption of the CM dose to the specific ATVS slider and reference setting.

In conclusion, the proposed approach of CTA scan protocol optimization is another step towards personalized medicine in radiology. It is based on a combination of ATVS settings and adapted CM injection protocols. Taking a state-of-the-art 90-kV exam as reference for thoracoabdominal CTA, the feasibility of a 26% reduction in CM dose or a 30% reduction in radiation dose without reducing qualitative and quantitative image quality was demonstrated in pigs. This method appears robust, and its efficacy has also been demonstrated under simulated obesity conditions.

## Data Availability

The datasets used and/or analyzed during the current study are available from the corresponding author on reasonable request.

## Abbreviations

ATVS: Automated tube voltage selection
CM: Contrast media
CNR: Contrast-to-noise ratio
CT: Computed tomography
CTA: CT angiography
CTDI_vol_: Volume-weighted computed tomography dose index
FID: Fréchet inception distance
PSNR: Peak signal-to-noise ratio
ROI: Region of interest
SD: Standard deviation
